# Diffuse pulmonary lesions caused by ANCA-associated vasculitis: A case report

**DOI:** 10.1097/MD.0000000000043811

**Published:** 2025-08-22

**Authors:** Huiying Chen, Zehui Lin, Xiaoyun Jian

**Affiliations:** a The Eighth Clinical Medical College of Guangzhou University of Chinese Medicine, Guangzhou, Guangdong, China; b Foshan Hospital of Traditional Chinese Medicine, Foshan, Guangdong, China.

**Keywords:** ANCA, ANCA vasculitis, misdiagnose, pneumonia, vasculitis

## Abstract

**Rationale:**

Anti-neutrophil cytoplasmic antibody–associated vasculitis (AAV) encompasses rare, multisystem autoimmune diseases such as granulomatosis with polyangiitis (GPA), microscopic polyangiitis, and eosinophilic GPA. This group of vasculitides can manifest at any age, affecting various organ systems, with a notable frequency of respiratory involvement in GPA and microscopic polyangiitis. Pulmonary symptoms, often similar to those of respiratory infections, frequently complicate the diagnosis. Lesions associated with alveolar hemorrhage are challenging to identify on imaging, leading to potential delays in AAV diagnosis and treatment. This case highlights the diagnostic challenges and the importance of early multidisciplinary collaboration in diagnosing AAV.

**Patient concerns:**

A middle-aged female presented with a 2-month history of recurrent cough and sputum production that did not improve with antibiotics. Initial chest computed tomography (CT) revealed multiple areas of increased density in both lungs, initially suspected as pneumonia.

**Diagnoses:**

Persistent symptoms despite antibiotic therapy, a positive anti-myeloperoxidase (MPO-IgG) antibody test, and concurrent sinusitis raised suspicion for AAV, which was confirmed through multidisciplinary consultation.

**Interventions:**

The patient was treated with prednisone and azathioprine.

**Outcomes:**

Symptomatic improvement was noted within 1 week of treatment. Follow-up chest CT at 3 months showed complete resolution of pulmonary lesions.

**Lessons:**

This case underscores the diagnostic challenges of AAV, especially when respiratory symptoms and CT findings resemble an infection. In patients with unexplained respiratory symptoms and diffuse lung changes unresponsive to antibiotics, AAV should be part of the differential. Early anti-neutrophil cytoplasmic antibody testing and multidisciplinary collaboration are crucial for timely diagnosis and treatment.

## 1. Introduction

Anti-neutrophil cytoplasmic antibody (ANCA)-associated vasculitis (AAV) is a group of rare multisystem autoimmune vasculitis, including granulomatosis with polyangiitis (GPA), microscopic polyangiitis (MPA) and eosinophilic granulomatosis with polyangiitis (EGPA) (formerly known as Churg–Strauss syndrome). ANCAs are autoantibodies targeting neutrophil cytoplasmic antigens. They are typically classified by immunofluorescence into 2 patterns: cytoplasmic ANCA, which mainly corresponds to proteinase 3, and perinuclear ANCA, which primarily targets myeloperoxidase (MPO-ANCA). These patterns have important diagnostic value: proteinase 3-ANCA is most commonly associated with GPA, while MPO-ANCA is more frequently observed in MPA and EGPA.^[1]^ This disease can occur at any age and affects all races.^[2,3]^ Recent studies report prevalence rates of 300 to 421 per million people.^[4,5]^ AAV can affect multiple organ systems, with a wide range of clinical manifestations. More than half of patients with GPA and one-third of those with MPA have lower respiratory tract involvement.^[6,7]^ Up to 30% of GPA cases are limited to the upper or lower respiratory system.^[8]^ The manifestations of AAV involving the lower respiratory tract often resemble respiratory infections, particularly exudative lesions caused by alveolar hemorrhage. These lesions are frequently difficult to detect on chest X-rays and challenging to differentiate from lung infections on computed tomography (CT) scans, often resulting in delayed diagnosis. This presents a significant challenge for clinicians in diagnosing and treating AAV. Current literature highlights several gaps in the diagnosis and management of AAV. First, there is a lack of standardized diagnostic protocols for patients presenting with atypical respiratory symptoms. Second, the role of multidisciplinary collaboration in early diagnosis has not been sufficiently explored. Third, while ANCA testing is a cornerstone of AAV diagnosis, its integration into routine clinical practice for patients with unexplained respiratory symptoms remains inconsistent. This article introduces a patient who came to the clinic with respiratory symptoms and diffuse lung changes observed on lung CT and was quickly diagnosed with AAV.

## 2. Case presentation

**Figure 1.**
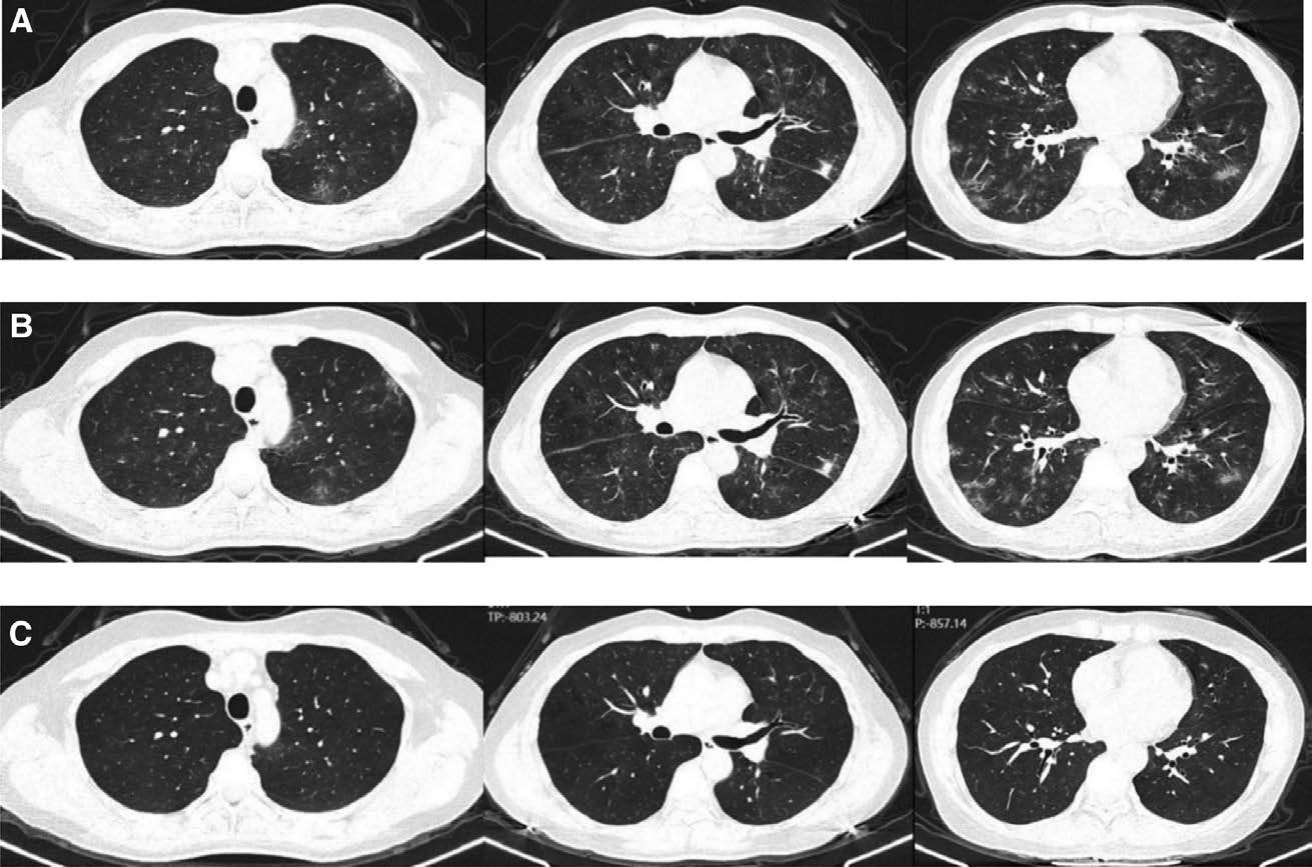
(A) Initial chest CT on admission: multiple scattered areas of increased density in both lungs. (B) A follow-up chest CT 1 week later: no improvement in lung lesions. (C) A chest CT scan performed 3 months: full resolution of the lung lesions. CT = computed tomography.

A middle-aged female office worker presented to the clinic in November 2023 with a 2-month history of recurrent cough and sputum production without an identifiable cause. The patient’s clinical symptoms included cough and sputum production, occasionally accompanied by a small amount of blood in the sputum. She denied fever, chest pain, dyspnea, rash, joint pain, and reported occasional nasal congestion. Vital signs were stable. Laboratory tests showed a hemoglobin concentration of 70 g/L, an erythrocyte sedimentation rate of 55 mm/h, and normal values for white blood cell count, high-sensitivity C-reactive protein, creatinine, liver function, and urinalysis results. Initial chest CT on admission revealed multiple scattered areas of increased density in both lungs (Fig. 1). The patient was initially diagnosed with pneumonia, and bronchoscopy with alveolar lavage fluid collection was performed. Metagenomic pathogen gene sequencing of the alveolar lavage fluid specimen detected *Haemophilus influenzae*. Although metagenomic testing identified *Haemophilus influenzae*, no formal differential cell count was documented, and hemosiderin-laden macrophages were not reported due to the institutional limitation that routine cytological classification is not performed in our bronchoscopy unit. This limitation may have affected the ability to confirm diffuse alveolar hemorrhage. After 1 week of moxifloxacin therapy, her symptoms did not significantly improve. A follow-up chest CT 1 week later showed no improvement in lung lesions (Fig. 1). Complete sinus CT imaging revealed sinusitis, and anti-myeloperoxidase IgG (MPO-IgG) antibody was positive. After thorough consultation with a rheumatologist, a likely diagnosis of AAV was established. Treatment was initiated with prednisone 55 mg daily, reduced by 5 mg every 2 weeks, combined with azathioprine 100 mg daily. During follow-up at the rheumatology clinic 1 week posttreatment, the patient reported significant improvement in cough and sputum symptoms. At her 1-month follow-up, respiratory symptoms had completely resolved. A chest CT scan performed 3 months after treatment (Fig. 1) showed full resolution of the lung lesions.

## 3. Discussion

The initial symptoms of AAV commonly include fever, joint pain, hematuria, and fatigue. More than 50% of patients present with cumulative lung involvement, particularly in cases of EGPA and GPA. Typical clinical manifestations include cough, sputum production, hemoptysis, and respiratory failure. The primary CT findings include interstitial lung disease (ground-glass opacities, honeycomb changes, reticular patterns), granulomas (nodules, masses, with or without cavitation), alveolar hemorrhage, and airway involvement (bronchiectasis, bronchial stenosis).^[6,7,9]^ MPA and GPA patients demonstrate distinct patterns of pulmonary involvement: cavitary nodules are more frequently seen in GPA, while pulmonary fibrosis is more common in MPA. Chest CT findings in both diseases may show ground-glass opacities and diffuse alveolar hemorrhage.^[9]^ A Spanish survey reported an average diagnostic delay of up to 3 months for these patients, with about 6% experiencing a delay exceeding 1 year.^[7]^ Accurately diagnosing AAV, assessing disease severity, and distinguishing between active vasculitis, infection, and other complications or comorbidities remains challenging.^[10]^ Although MPO-ANCA is found in both MPA and EGPA, several clinical features distinguish the 2 conditions. EGPA is typically associated with a history of asthma and peripheral eosinophilia. In contrast, our patient had no asthma or eosinophilia and did not exhibit allergic manifestations.^[11–13]^ These findings, combined with the pulmonary symptoms and positive MPO-ANCA, make MPA the more likely diagnosis. The patient described in this article presented with respiratory symptoms without fever, fatigue, upper respiratory symptoms, or renal impairment. Her CT scan showed diffuse ground-glass opacities, solid nodules, and patchy areas with indistinct margins and variable density in both lungs, suggesting AAV involvement. Based on imaging, the nodules were diffusely and randomly distributed throughout both lungs, without a specific peribronchial or subpleural predominance. This pattern is consistent with a random distribution, which is typically associated with hematogenous spread and is observed in certain infectious or vasculitic conditions. After 1 week of antibiotic therapy, we determined that the lung changes observed on CT likely represented alveolar hemorrhage rather than infection. The presence of concurrent sinusitis, positive MPO-IgG antibody, and clinical symptoms consistent with AAV allowed a clinical diagnosis without tissue biopsy.

We performed a search on PubMed using the following keywords: “Anti-neutrophil cytoplasmic antibody-associated vasculitis,” “ANCA-associated vasculitis,” “ANCA,” “granulomatosis with polyangiitis,” “microscopic polyangiitis,” “eosinophilic granulomatosis with polyangiitis,” and “lung.” Based on the case reports retrieved, we observed that AAV can present with isolated respiratory symptoms, coexist with other systemic diseases, or manifest as a single symptom in other organ systems. AAV can involve multiple systems and organs, including the heart, kidneys, brain, uterus, nervous system, ears, eyes, liver, gastrointestinal tract, oral cavity, and hematopoietic system, with the lungs and kidneys being the most frequently reported sites of involvement. AAV can be either primary or secondary to other conditions, such as COVID-19, interstitial pneumonia, idiopathic pulmonary fibrosis, renal diseases, or drug-induced cases. AAV is often misdiagnosed as organizing pneumonia,^[1]^ lung cancer,^[2]^ or pulmonary tuberculosis,^[3]^ among other respiratory diseases. Notably, some patients test negative for ANCA-related markers and are ultimately diagnosed with AAV through biopsy. In our review, we identified 11 cases of AAV in which patients were initially diagnosed with pneumonia, showed no improvement after antibiotic therapy, and were subsequently confirmed as AAV, primarily through biopsy. Table 1 summarizes the general characteristics of these patients and the features of AAV.^[14–25]^

**Table 1:** General characteristics of ANCA-associated vasculitis patients misdiagnosed as pneumonia.

References	Sex	Age	ANCA	Vasculitis classification	Lungs	Kidney	ENT	Eyes	Skin	Joints	Heart
Fan et al^[14]^	F	63	p-ANCA+, anti-MPO+	AAV renal injury	Yes	Yes	–	–	–	–	–
Bande et al^[15]^	F	35	c-ANCA+	Wegener granulomatosis	Yes	Yes	Yes	–	–	–	–
Theodorou et al^[16]^	F	76	–	Wegener granulomatosis	Yes	–	–	–	–	–	–
Laube and Thurnheer^[17]^	F	65		Eosinophilic granulomatosis with polyangiitis	Yes	–	–	–	–	–	–
Mroz et al^[18]^	F	50	–	Churg–Strauss syndrome	Yes	–	–	–	Yes	–	–
Rodrigues et al^[19]^	F	55	Anti-PR3+	Granulomatosis with polyangiitis	Yes	Yes	Yes	Yes	Yes	–	–
Berthoux et al^[20]^	M	54	Anti-PR3+	Wegener granulomatosis	Yes	Yes	–	–	–	–	–
Rosmarakis et al^[21]^	M	35	c-ANCA+	Wegener granulomatosis	Yes	–	Yes	–	–	–	–
Ge et al^[22]^	F	56	anti-PR3+	Granulomatosis with polyangiitis	Yes	–	Yes	–	–	–	–
Zhang et al^[23]^	M	40s	–	ANCA-negative pulmonary vasculitis or a rare case of IgA-associated pulmonary capillaritis	Yes	Yes	Yes	–	–	–	–
Despond et al^[24]^	F	56	–	Microscopic polyangiitis	Yes	Yes	–	–	–	–	–
Chippa et al^[25]^	F	54	anti-PR3+	Granulomatosis with polyangiitis	Yes	–	–	–	–	–	–

AAV = antibody-associated vasculitis, ANCA = anti-neutrophil cytoplasmic antibody, c-ANCA = cytoplasmic ANCA, MPO = myeloperoxidase, p-ANCA = perinuclear ANCA, PR3 = proteinase 3.

Our case demonstrates that early ANCA testing and a high index of suspicion can significantly reduce the delay in diagnosis. This approach not only improves patient outcomes but also reduces the risk of complications associated with delayed treatment. Furthermore, this case underscores the importance of differentiating AAV from infections. The initial misdiagnosis of pneumonia highlights the need for clinicians to consider AAV in patients with atypical respiratory presentations, particularly when antibiotic therapy fails to improve symptoms. Future studies should explore the role of multidisciplinary teams in improving the diagnostic accuracy and management of AAV.

## 4. Conclusion

AAV frequently affects the lungs, with diverse clinical symptoms and CT imaging manifestations, and is often complicated by infection, making it difficult to distinguish from lung infection. For patients with unexplained respiratory symptoms and persistent diffuse lung changes unresponsive to antibiotics, AAV should be considered. Prompt diagnosis and treatment are essential to prevent disease progression, underscoring the importance of comprehensive clinical and laboratory assessment in patients with atypical respiratory presentations. This case illustrates the diagnostic challenges and the value of early ANCA testing and multidisciplinary collaboration. Unlike previous reports, our patient was rapidly diagnosed through a combination of clinical suspicion, positive MPO-IgG antibody, and imaging findings, leading to prompt treatment and resolution of symptoms. This case adds to the literature by demonstrating the importance of a high index of suspicion for AAV in patients with persistent respiratory symptoms and the role of multidisciplinary teams in achieving an accurate diagnosis.

## Author contributions

**Conceptualization:** Zehui Lin, Xiaoyun Jian.

**Data curation:** Huiying Chen.

**Formal analysis:** Huiying Chen.

**Project administration:** Xiaoyun Jian.
